# Older Adults’ Pain Outcomes After mHealth Interventions: Scoping Review

**DOI:** 10.2196/46976

**Published:** 2023-05-31

**Authors:** Marcia Shade, Mariya Kovaleva, Kimberly Harp, Aqueasha Martin-Hammond

**Affiliations:** 1 College of Nursing University of Nebraska Medical Center Omaha, NE United States; 2 Luddy School of Informatics, Computing, and Engineering Indiana University-Purdue University Indianapolis, IN United States

**Keywords:** mHealth, older adults, pain, self-management, pain management, mobile health, musculoskeletal pain, scoping review, pain outcomes, mobile phone

## Abstract

**Background:**

Pain is prevalent and poorly managed in older adults. Although pain self-management strategies are helpful, adoption and access are limited; thus, technology provides an opportunity for intervention delivery. Mobile health (mHealth) is feasible to use in older adults; however, we have yet to understand the effect of mHealth pain self-management interventions on pain outcomes in older adults.

**Objective:**

The purpose of this scoping review is to examine the characteristics of mHealth interventions and their efficacy on pain outcomes in older adults with musculoskeletal pain.

**Methods:**

With the assistance of a medical librarian, keywords and subject headings were generated, including but not limited to mobile health application, mHealth, digital, pain, pain management, and older. A search was conducted for papers in journal databases, including PubMed, Embase, CINAHL, Scopus, and IEEE Xplore, between 2000 and 2022. Papers were screened according to predetermined inclusion and exclusion criteria, and reference lists were reviewed for additional paper inclusion. Three authors appraised the methodology of papers independently, then collaboratively to synthesize the evidence.

**Results:**

Six publications were included in the scoping review. The design and methodology ranged widely from pilot studies to a comparative effectiveness trial. Older participants in the studies reported a variety of musculoskeletal conditions. Delivery of the mHealth pain self-management interventions incorporated mobile devices, such as a smartphone or tablet. Most mHealth-delivered interventions were multicomponent and incorporated elements of in-person and telephone access to an interventionist. The findings suggested mHealth interventions may reduce pain intensity; however, pain interference and other pain-related conditions did not have a statistically significant reduction.

**Conclusions:**

Research that has explored mHealth for pain self-management is beginning to move beyond feasibility. The few experimental studies conducted in older adults are heterogeneous, and the interventions are mostly multicomponent. It is premature to conclude the interventions’ significant effect on pain or pain-related symptoms. As technology continues to integrate into health care, more experimental research is warranted to examine the efficacy of mHealth interventions on a variety of pain outcomes in older adults.

## Introduction

The population of older adults in the United States is expected to reach 80.8 million by 2040. Along with the population increase, it is likely that the number of individuals with chronic conditions and symptoms, such as chronic pain, will grow [1]. The prevalence of pain in adults 65 years of age and older is higher than in the general adult population globally and in the United States [2].

The concept of “pain self-management” has not been well-defined in the literature. Some researchers have defined self-management as a behavior that helps patients maintain wellness through several tasks: condition management, creation of new meaningful behaviors, and emotion management [3]. Researchers have also postulated that the term “self-management” is often used interchangeably with terms such as “self-help,” “guided self-help,” “self-management strategies,” “self-management interventions,” “coping strategies,” and “self-care.” It remains unclear whether pain self-management presumes a collection of strategies for patients or packaged interventions and treatments [4]. Since pain self-management has not been consistently defined in the literature, this variation can influence studies exploring this concept.

Pain self-management has been historically encouraged among older adults with chronic pain [3,5]. For example, yoga, tai chi, qigong, massage, cognitive behavioral therapy, and music therapy are self-management interventions that have been reported to improve musculoskeletal pain in older adults [5-7]. To improve accessibility and adoption, pain self-management interventions have also been developed and delivered using technology. In fact, mobile health (mHealth) software apps are a modality that has gained popularity to promote pain self-management interventions [8-10].

While definitions of mHealth vary, it is often defined as the use of mobile phones, tablets, sensors, and other wireless devices to help accomplish health objectives [11,12]. mHealth software apps are typically task-specific, health-related computer programs that can be downloaded on the internet for use on a personal mobile device. Not only may mHealth apps track health and lifestyle [13], but these apps may also facilitate symptom and disease management among older adults [14]. In the pain realm, the function of mHealth may support an older adult’s remote pain monitoring and reporting, improve patient–health care provider communication, and promote therapeutic pain treatment delivery and pain research access [15].

The possibilities are numerous for mHealth and older adults who have pain. In total, 28% of older adults currently use at least one mHealth app, and 49% of those between the ages of 50 and 64 years were more likely to have used an app than 38% of older adults between 65 and 80 years of age [16]. Although there are usability barriers with devices that deliver mHealth, pain management via mHealth is feasible and acceptable for older adults [17]. Among older adults with minimal prior use of mHealth, most report willingness to try mHealth to help manage pain symptoms and pain medications. Older adults also appreciate the potential benefit of being able to contact their health care provider and safely manage their pain [18]. Given the acceptability and potential usefulness of mHealth pain self-management interventions for older adults, a review of the evidence is warranted. This scoping review was guided by the following research questions: What are the characteristics of the mHealth pain interventions that have been used in older adults? What effect do mHealth interventions have on pain outcomes in older adults?

## Methods

### Study Design

The aim of a scoping review is to conduct a broad analysis of available evidence to answer a research question [19] and demonstrate the range of evidence pertinent to the research question [20]. The methodology used in this review is based on the Arksey and O’Malley [19] framework. This scoping review involved five steps: (1) formulating the research questions, (2) identifying relevant studies, (3) study selection, (4) charting the data, and (5) collating, summarizing, and reporting the results [19,20].

### Search Strategy

To obtain a comprehensive review of the literature, the research team performed a search across multiple bioscience and biomedical databases, including MEDLINE via PubMed, Embase, CINAHL, Scopus, and IEEE Xplore for pertinent papers.  The results were limited to peer-reviewed papers published in English between 2000 and 2022. The search was performed in accordance with the PRISMA (Preferred Reporting Items for Systematic Reviews and Meta-Analyses) guidelines. The search strategy, designed by an experienced academic medical librarian (KH), combined controlled vocabulary terms and free-text words on the concepts of mobile apps, pain management, and adults aged 60 years or older. The full search strategy is included in Multimedia Appendix 1. The systematic search was last run in September 2022. To minimize bias, the researchers used a broad search strategy to be inclusive across gender, sex, orientation, race, ethnicity, ability, literacy, socioeconomic status, and comorbidity. We solely focused on interventions for noncancer chronic pain among older adults. The search resulted in 1840 unique papers. The research team used the citation manager, RefWorks, to remove 4 duplicate papers. This resulted in 1838 unique papers. These papers were then screened and ascertained for relevance to the inclusion and exclusion criteria.

### Eligibility Criteria

**Figure 1:**
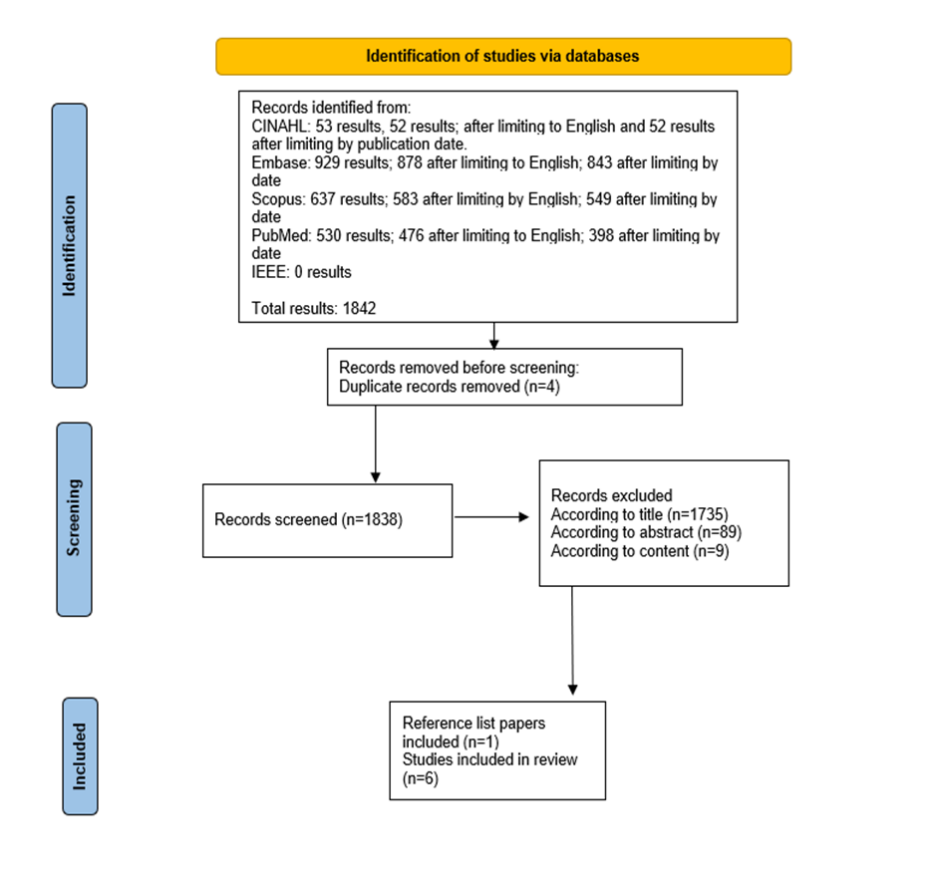
Scoping review flowchart mHealth interventions for pain outcomes in older adults.

Studies were included in this review if they aimed to explore the outcomes of using mHealth pain interventions in older adults. Older adults were defined as those individuals 60 years of age or older as characterized by the World Health Organization [21]. Studies were included if the mean age of study participants was 60 years or older. We included intervention and cohort studies published in English. Studies were excluded if the pain intervention was delivered on a personal desktop computer (ie, nonmobile device) or if participants did not report musculoskeletal pain. Study protocols, opinion and editorial papers, reviews, case studies, abstracts, position statements, dissertations and theses, guidelines, book chapters, conference proceedings, and solely qualitative studies were excluded. The flowchart [22] of the database search and screening process is shown in Figure 1. Papers were evaluated according to study methodology.

### Charting the Data

Three authors (MS, AMH, and MK) selected the final set of studies. Two authors (MS and MK) reviewed and organized the data that pertained to research questions. The data included country, median or mean age, sample size, race or ethnicity, clinical pain diagnoses or conditions that were the subject of treatment, devices used to deliver mHealth, mHealth intervention characteristics, and pain outcomes. Six papers were included, and the data are summarized in Multimedia Appendix 2 [23-47].

## Results

### Overview

In total, 4 of the selected studies were conducted in the United States, 1 study was conducted in Sweden, and 1 in the United Kingdom. The study designs varied; 2 studies were randomized controlled trials [48,49]. Other designs included a mixed methods exploratory within-subject pre- and posttest study [50], longitudinal cohort study [51], a phased randomized wait list control trial [52], and a randomized noninferiority comparative effectiveness trial [53].

The papers included men and women (mean age range 63-70 years; 45%-91.7% White). Sample sizes of the studies ranged from 12 [50] to 499 participants [51]. The study participants’ clinical pain diagnoses included those commonly associated with chronic musculoskeletal pain. Pain and corresponding diagnoses included knee or hip pain from osteoarthritis [48,51], back pain [53], multiple pain locations [52], musculoskeletal and neurological pain [50], and chronic noncancer pain [49].

### Devices Used for Intervention Delivery

In 3 studies, a smartphone or tablet were used to deliver a downloaded software app or stream content as an mHealth intervention [48,49,51]. Another study incorporated a wrist-worn activity monitor with a software app delivered using a smartphone or tablet [52]. One study incorporated an artificial intelligence engine that delivered the mHealth intervention using an interactive voice response via telephone [53]. One study used a virtual reality (VR) headset to deliver the mHealth intervention [50].

### mHealth Pain Self-management Intervention Characteristics

Despite the similarity in using mHealth, the interventions varied widely in the structure, content, and duration. Overall, the structure of the intervention components varied. Interventions incorporated a mixture of mHealth along with in-person and access to other interventionalists such as a coach or therapist. An in-person component was present in one study [52]. By contrast, in another study [52], an initial phase of the intervention included group sessions led by interventionists. In 4 studies, telephone access to an interventionist was offered [48,49,51,53].

The studies also varied in content and duration. In 2 studies, exercise and osteoarthritis education were the main intervention components [48,51]. In the VR study, distraction was used for coping and relaxation as the main intervention component. Older adults played games or interacted for 15-45 minutes, with virtual activities focused on pet engagement, animals, music, and travel [50]. In one study, pain and coping skills training were used. The pain and coping skills training was web-based and included group videoconferencing led by expert facilitators. An app was used as part of the intervention to help tailor pain management goals for 12 weeks. Although it was in the first 3 weeks of the intervention, this was the only study with in-person groups [52]. One study included a multicomponent intervention to encourage behavioral activities for symptom monitoring. Behavioral activities included the daily entry of pain symptoms, diet, and behavior tracking. Older adults received weekly health telecoaching sessions with tailoring of lifestyle adjustments to manage pain symptoms [49]. One study used cognitive behavioral therapy delivered by artificial intelligence, and the intervention incorporated daily interactive voice feedback [53].

### Pain and Pain-Related Outcomes

In each study, the intervention’s efficacy for decreasing pain intensity was measured. In 5 studies [48-51,53], pain intensity was measured using the numeric rating scale [23,27]. Fanning and colleagues [52] used the Patient-Reported Outcomes Measurement Information System (PROMIS) 3-item pain intensity scale [29]. In 2 studies where the numeric rating scale was used, pain intensity was measured within the past week [48,51]. In one study, current pain intensity was measured [49]. In 2 studies, a time-framed measure of the pain outcome was undefined [50,53]. In the study that used the PROMIS tool, pain intensity was measured in the previous week [52]. A decrease in pain intensity after the delivery of the mHealth interventions was reported in all studies.

Pain interference was measured in 2 studies. In one study [53], the items on the Brief Pain Inventory were used [44,45], and in another study [52], the PROMIS pain interference scale was used [29]. A statistically significant difference was not found in pain interference after the use of an mHealth intervention in one study [52]. In the other study [53], it was not reported whether pain interference had a statistically significant change after the mHealth intervention.

Additional pain-related outcomes were measured in the selected papers. Depressive symptoms were measured in 2 studies [50,53] and did not improve after the mHealth interventions. A nonsignificant decrease in anxiety and emotional affect was found in one study [49]. Similarly, in the 2 studies that measured quality of life, no change was found [49,50]. Pain-related disability was measured in 2 studies and did not demonstrate a statistically significant improvement after implementing mHealth interventions [49,53].

## Discussion

### Principal Findings

A scoping review was conducted to examine the characteristics of mHealth interventions and their efficacy on pain outcomes in older adults. Consistent with the purpose of a scoping review, we demonstrated the breadth of evidence on a topic [19]. The main findings of our review were (1) mHealth interventions varied widely in structure, content, duration, and target audience in terms of pain diagnoses; (2) there was a statistically significant reduction in pain intensity in all studies; and (3) varied results were attained regarding other pain-related outcomes, with no consensus possible, due to the small number of studies to date.

The number of studies that focus on measuring pain or pain-related outcomes in older adults is sparse. Most studies where mHealth for pain was explored were excluded due to participants having a mean age of less than 60 years old. The sample sizes of the studies varied, which may be attributed to usability barriers in the oldest old of adults, leading to challenges in recruiting older participants for mHealth pain intervention studies. Many excluded studies also focused on usability, acceptance, and design of mHealth pain interventions, and yet while important for aiding adoption, many of these studies did not measure an effect on pain outcomes.

There were a variety of devices used to deliver mHealth interventions, including smartphones or tablets. The use of smartphones would be the most logical device to deliver mHealth interventions due to the availability and increased use by older adults. Despite the common assumption that older adults do not use technology, many have adopted mobile devices, which is consistent with Pew research describing older adult’s use of smartphones has grown [54]. VR is gaining momentum for use in treatment and intervention delivery. VR has not routinely been included in mHealth interventions; however, a wireless VR headset (with or without a smartphone) may be transformed for a therapeutic treatment by virtue of the intended use of the software it is running [55]. Thus, VR was included in this review based on the definition of mHealth used in this scoping review and to explore the broad range of evidence. Although VR was used in one study [50], most of the literature on VR is focused on factors related to attitudes and usability in older adults [56,57], as opposed to the effect on pain outcomes. Other emerging devices, such as smart speakers (eg, Amazon Alexa and Google Home) and wearables (eg, Apple Watch and Fitbit), have yet to be widely explored for the delivery of mHealth pain interventions.

Most pain self-management strategies incorporate content elements of pain education, training or coaching to strengthen relaxation skills, coping, problem-solving, and communication [5]. In this review, the mHealth pain interventions varied and combined multiple approaches to affect older adults’ pain. The structure of the interventions varied, with most interventions incorporating access to in-person [52] or telephone or chat [48,49,51,53] interventionist. One perspective on this incorporation of a human component to mHealth interventions is that older adults may be unable or unwilling to partake in mHealth interventions remotely without access to an interventionist. It may be necessary to include interventionist access to help ease anxiety, assist older adults, or ensure proper intervention delivery. It is also fair to propose that mHealth delivery can be used to complement in-person care or support traditional interventions (eg, offer mHealth intervention between traditional in-person physician’s appointments). The characteristics of the interventions discussed in the studies are consistent with the evidence associated with pain management strategies that need to be promoted in older adults. A multimodal approach to pain is encouraged to manage pain in older adults, and what works for one person may not work for someone else. Although it is unclear from the review, mHealth interventions may need to incorporate a variety of strategies for tailoring and tangible support for older adults.

Pain is a biopsychosocial symptom, and several instruments were used to assess pain outcomes. Pain intensity was a universal outcome, but measurement occurred at different time points. For example, it is not clear if mHealth interventions affect the older adult’s current pain intensity or pain that occurred within the past week. Surprisingly, pain interference was only measured in 2 studies [52,53] and did not improve. Pain interference is defined as the degree to which pain prevents an individual’s participation in physical, cognitive, emotional, sleep, recreational activities, and experiencing enjoyment [58]. Contingent on the evidence presented in this review, even if pain intensity decreases, pain may continue to interfere with aspects of the older adult’s life. Therefore, a reduction in pain intensity may not capture an outcome that is meaningful to patients, particularly if pain continues to hinder activities. Self-reported pain interference needs to be measured in future studies using mHealth interventions for pain in older adults. Also, based on the heterogeneity and small number of studies in this review, it is unclear whether incorporating mHealth pain interventions significantly affects pain-related conditions, such as mental and emotional symptoms and disability. Future studies need to measure the biopsychosocial elements of pain and consistently use validated and reliable instruments to give direction and support for interventions.

More experimental studies are needed with large and culturally diverse samples to examine the efficacy of mHealth pain interventions on pain outcomes. Recruitment strategies must be such that older adults will be assured that training and assistance with technology will be provided to help alleviate apprehensions associated with usability. One example of assistance would be “navigators” that help older adults become acclimated with and troubleshoot the technology [59,60]. It is important to continue examining the role and outcomes of mHealth interventions for pain management in older adults. With the growing adoption of mobile devices, older adults have greater opportunities to use mHealth for pain management as a nonpharmacologic strategy, which may improve health outcomes and quality of life. Additionally, pragmatic issues of mHealth use among older adults such as cost and internet access may be examined.

### Limitations

There are some limitations in this scoping review. The search identified few papers that focused on mHealth interventions for pain in older adults. As this is a rapidly growing body of research, other relevant papers could have been missed. For example, we did not include conference proceedings or abstracts from human-computer interaction databases, which may include additional experiments of mHealth pain interventions in older adults. The mental and behavioral health database, PsycInfo, was eliminated during the medical librarian’s preliminary search due to zero returns on the search terms combining keywords and controlled vocabulary for the concepts of mobile apps and chronic pain. Publication bias may have also caused us to miss papers with a negative or poor pain outcomes associated with mHealth interventions. Additionally, we only selected papers published in English.

### Conclusions

This scoping review was conducted to examine the characteristics of mHealth interventions and pain outcomes in older adults. Although the research is beginning to move beyond usability and acceptance, few experimental studies have been conducted in older adults that focus on improving pain outcomes. The studies that have been conducted vary in design, sample size and diversity, measured outcomes, and interventional approach. As technology continues to integrate into health care, more research is warranted to examine the efficacy of mHealth interventions on pain outcomes in older adults.

## Appendix

Multimedia Appendix 1 Search strategy.

Multimedia Appendix 2 Mobile health intervention characteristics and outcomes.

## Abbreviations

mHealth: mobile health
PRISMA: Preferred Reporting Items for Systematic Reviews and Meta-Analyses
PROMIS: Patient-Reported Outcomes Measurement Information System
VR: virtual reality
