# Supplementary material for: Older Adults’ Pain Outcomes After mHealth Interventions: Scoping Review
Source: JMIR Aging. 2023 May 31;6:e46976. doi: 10.2196/46976 (PMC10267779; doi:10.2196/46976)
Supplement: Multimedia Appendix 1 [file aging_v6i1e46976_app1.docx]

Comprehensive search strategies (KH)

*CINAHL search strategy:*

"mobile health application" OR "mobile apps" OR "mobile application" OR "health app" OR "health mobile applications" OR "health care app" OR "health care applications" OR “smartphone app” OR “smartphone applications” OR “mobile medical apps” OR “mobile medical applications” OR cellphone* OR ( mhealth AND applications ) OR ( ehealth AND applications ) OR  (MH "Mobile Applications") OR tablet* OR “voice assistant” OR “digital assistant” OR “digital assistants” OR “virtual assistant” OR Alexa OR Siri OR “smart watch” OR “smart watches” OR smartwatch* OR “fitness tracker” OR “fitness trackers” OR “activity tracker” OR “digital health device” OR “wearable devices” OR “digital health technology” OR “digital health technologies” OR “wireless medical device”

AND

“chronic pain” OR “chronic pain management” OR “pain management” OR “chronic pain” OR “pain syndrome OR “chronic pain syndrome” OR ((pain* OR “pain free” OR pain-free) AND (manage OR management)) OR (MH "Chronic Pain") OR “persistent pain”

AND

Aged OR elder* OR elderly OR “senior citizen” OR senior* OR “oldest old” OR “community dwelling” OR geriatric* aeging OR aging OR 65+ OR 55+ OR retire* OR retiree* OR pensioner* OR “nursing home” OR “nursing homes” OR “senior center” OR “senior centers” OR “assisted living” OR older OR centenarian* OR octogenarian* OR nonagenarian* OR septuagenarian* OR sexagenarian* OR grandparent* OR grandmother* OR grandfather* OR grandpa* OR grandma* OR gerontol* OR medicare OR “55 years” OR “55-years” OR “60-years” OR “60 years” OR "65 years" OR "65-years" OR "60-years" OR "70 years" OR "70-years" OR "75 years" OR "75-years" OR "80 years" OR "80-years" OR "85 years" OR "85-years" OR "90 years" OR "90-years" OR "95 years" OR "95-years" OR "100 years" OR "100-years" OR"60 year" OR "65 year" OR "65-year" OR "60-year" OR "70 year" OR "70-year" OR "75 year" OR "75-year" OR "80 year" OR "80-year" OR "85 year" OR "85-year" OR "90 year" OR "90-year" OR "95 year" OR "95-year" OR "100 year" OR "100-year" OR "sixty years" OR "sixty-years" OR "sixty-five years" OR "sixty-five-years" OR "seventy years" OR "seventy-years" OR "seventy-five years" OR "seventy-five-years" OR "eighty years" OR "eighty-years" OR "eighty-five years" OR "eighty-five-years" OR "ninety years" OR "ninety-years" OR "ninety-five years" OR "ninety-five-years" OR "hundred years" OR "hundred-years" OR "hundred years" OR "hundred-years" OR "one-hundred years" OR "one-hundred-years" OR "sixty year" OR "sixty-five year" OR "sixty-five-year" OR "sixty-year" OR "seventy year" OR "seventy-year" OR "seventy-five year" OR "seventy-five-year" OR "eighty year" OR "eighty-year" OR "eighty-five year" OR "eighty-five-year" OR "ninety year" OR "ninety-year" OR "ninety-five year" OR "ninety-five-year" OR "hundred year" OR "hundred-year" OR "one-hundred year" OR "one-hundred-year" OR (MH "Aged+") OR (MH "Gerontologic Care") OR (MH "Gerontologic Nursing+") OR (MH "Housing for the Elderly") OR (MH "Senior Centers") OR (MH "Aged, Hospitalized") OR (MH "Health Services for the Aged") OR (MH "Aged, 80 and Over+") OR (MH "Centenarians") OR (MH "Frail Elderly")

*Embase search strategy:*

('mobile application'/exp/mj OR 'mobile health application'/exp OR 'mobile health application' OR 'mobile apps'/exp OR 'mobile apps' OR 'mobile application'/exp OR 'mobile application' OR 'health app' OR 'health mobile applications' OR 'health care app' OR 'health care applications' OR 'smartphone app' OR 'smartphone applications' OR (cellphone* AND applications) OR (mhealth NEAR/4 applications) OR (('mhealth'/exp OR mhealth) AND applications) OR (ehealth NEAR/4 applications) OR (('ehealth'/exp OR ehealth) AND applications) OR 'mobile medical apps' OR 'mobile medical applications' OR tablet* OR 'voice assistant' OR 'digital assistant' OR 'digital assistants' OR 'virtual assistant' OR alexa OR siri OR 'smart watch' OR 'smart watches' OR smartwatch* OR 'fitness tracker' OR 'fitness trackers' OR 'activity tracker' OR 'digital health device' OR 'wearable devices' OR 'digital health technology' OR 'digital health technologies' OR 'wireless medical device')

AND

'chronic pain'/exp/mj OR 'chronic pain management' OR 'pain management' OR 'chronic pain' OR 'pain syndrome' OR 'chronic pain syndrome' OR ((pain* OR 'pain free') NEAR/3 (manage OR management))

AND

('aged'/exp OR aged OR elder* OR 'elderly'/exp OR elderly OR 'senior citizen'/exp OR 'senior citizen' OR senior* OR 'oldest old' OR 'community dwelling' OR 'geriatric* aeging' OR (geriatric* AND aeging) OR 'aging'/exp OR aging OR 65+ OR retire* OR retiree* OR pensioner* OR 'nursing home'/exp OR 'nursing home' OR 'nursing homes'/exp OR 'nursing homes' OR 'senior center'/exp OR 'senior center' OR 'senior centers'/exp OR 'senior centers' OR 'assisted living'/exp OR 'assisted living' OR older OR centenarian* OR octogenarian* OR nonagenarian* OR septuagenarian* OR sexagenarian* OR grandparent* OR grandmother* OR grandfather* OR grandpa* OR grandma* OR gerontol* OR 'medicare'/exp OR medicare OR '55 years' OR '65 years' OR '60 years' OR '70 years' OR '75 years' OR '80 years' OR '85 years' OR '90 years' OR '95 years' OR '100 years' OR '65 year' OR '60 year' OR '70 year' OR '75 year' OR '80 year' OR '85 year' OR '90 year' OR '95 year' OR '100 year' OR 'sixty years' OR 'sixty five years' OR 'seventy years' OR 'seventy five years' OR 'eighty years' OR 'eighty five years' OR 'ninety years' OR 'ninety five years' OR 'hundred years' OR 'one hundred years' OR 'sixty five year' OR 'sixty year' OR 'seventy year' OR 'seventy five year' OR 'eighty year' OR 'eighty five year' OR 'ninety year' OR 'ninety five year' OR 'hundred year' OR 'one hundred year')

*PubMed search strategy:*

"mobile applications"[MeSH Terms] OR ("mobile"[All Fields] AND "applications"[All Fields]) OR "mobile applications"[All Fields] OR ("mobile"[All Fields] AND "application"[All Fields]) OR "mobile application"[All Fields] OR "mobile health application"[All Fields] OR "mobile apps"[All Fields] OR "mobile application"[All Fields] OR "health app"[All Fields] OR "health mobile applications"[All Fields] OR "health care app"[All Fields] OR "health care applications"[All Fields] OR "smartphone app"[All Fields] OR "smartphone applications"[All Fields] OR "cellphone*"[All Fields] OR "mobile medical apps"[All Fields] OR "mobile medical applications"[All Fields] OR "tablet*"[All Fields] OR "voice assistant"[All Fields] OR "digital assistant"[All Fields] OR "digital assistants"[All Fields] OR "virtual assistant"[All Fields] OR "Alexa"[All Fields] OR "Siri"[All Fields] OR "smart watch"[All Fields] OR "smart watches"[All Fields] OR "smartwatch*"[All Fields] OR "fitness tracker"[All Fields] OR "fitness trackers"[All Fields] OR "activity tracker"[All Fields] OR "digital health device"[All Fields] OR "wearable devices"[All Fields] OR "digital health technology"[All Fields] OR "digital health technologies"[All Fields] OR "wireless medical device"[All Fields]

AND

"chronic pain"[All Fields] OR "chronic pain management"[All Fields] OR ("chronic pain"[MeSH Terms] OR ("chronic"[All Fields] AND "pain"[All Fields]) OR "chronic pain"[All Fields])

AND

"aged"[MeSH Terms] OR "aged"[All Fields] OR "elder*"[All Fields] OR ("aged"[MeSH Terms] OR "aged"[All Fields] OR "elderly"[All Fields] OR "elderlies"[All Fields] OR "elderly s"[All Fields] OR "elderlys"[All Fields]) OR "senior citizen"[All Fields] OR "senior*"[All Fields] OR "oldest old"[All Fields] OR "community dwelling"[All Fields] OR ("geriatric*"[All Fields] AND "aeging"[All Fields]) OR ("aging"[MeSH Terms] OR "aging"[All Fields] OR "ageing"[All Fields]) OR "65"[All Fields] OR "retire*"[All Fields] OR "retiree*"[All Fields] OR "pensioner*"[All Fields] OR "nursing home"[All Fields] OR "nursing homes"[All Fields] OR "senior center"[All Fields] OR "senior centers"[All Fields] OR "assisted living"[All Fields] OR ("older"[All Fields] OR "olders"[All Fields]) OR "centenarian*"[All Fields] OR "octogenarian*"[All Fields] OR "nonagenarian*"[All Fields] OR "septuagenarian*"[All Fields] OR "sexagenarian*"[All Fields] OR "grandparent*"[All Fields] OR "grandmother*"[All Fields] OR "grandfather*"[All Fields] OR "grandpa*"[All Fields] OR "grandma*"[All Fields] OR "gerontol*"[All Fields] OR ("medicare"[MeSH Terms] OR "medicare"[All Fields] OR "medicare s"[All Fields] OR "medicares"[All Fields]) OR “55 years” OR “55-years” OR “60-years” OR “60 years” OR "65-years"[All Fields] OR "65-years"[All Fields] OR "60-years"[All Fields] OR "70-years"[All Fields] OR "70-years"[All Fields] OR "75-years"[All Fields] OR "75-years"[All Fields] OR "80-years"[All Fields] OR "80-years"[All Fields] OR "85-years"[All Fields] OR "85-years"[All Fields] OR "90-years"[All Fields] OR "90-years"[All Fields] OR "95-years"[All Fields] OR "95-years"[All Fields] OR "100-years"[All Fields] OR "100-years"[All Fields] OR "60-year"[All Fields] OR "65-year"[All Fields] OR "65-year"[All Fields] OR "60-year"[All Fields] OR "70-year"[All Fields] OR "70-year"[All Fields] OR "75-year"[All Fields] OR "75-year"[All Fields] OR "80-year"[All Fields] OR "80-year"[All Fields] OR "85-year"[All Fields] OR "85-year"[All Fields] OR "90-year"[All Fields] OR "90-year"[All Fields] OR "95-year"[All Fields] OR "95-year"[All Fields] OR "100-year"[All Fields] OR "100-year"[All Fields] OR "sixty-years"[All Fields] OR "sixty-years"[All Fields] OR "sixty five years"[All Fields] OR "sixty five years"[All Fields] OR "seventy-years"[All Fields] OR "seventy-years"[All Fields] OR "seventy five years"[All Fields] OR "seventy five years"[All Fields] OR "eighty-years"[All Fields] OR "eighty-years"[All Fields] OR "eighty five years"[All Fields] OR "eighty five years"[All Fields] OR "ninety-years"[All Fields] OR "ninety-years"[All Fields] OR "ninety five years"[All Fields] OR "ninety five years"[All Fields] OR "hundred-years"[All Fields] OR "hundred-years"[All Fields] OR "hundred-years"[All Fields] OR "hundred-years"[All Fields] OR "one hundred years"[All Fields] OR "one hundred years"[All Fields] OR "sixty-year"[All Fields] OR "sixty five year"[All Fields] OR "sixty five year"[All Fields] OR "sixty-year"[All Fields] OR "seventy-year"[All Fields] OR "seventy-year"[All Fields] OR "seventy five year"[All Fields] OR "seventy five year"[All Fields] OR "eighty-year"[All Fields] OR "eighty-year"[All Fields] OR "eighty five year"[All Fields] OR "eighty five year"[All Fields] OR "ninety-year"[All Fields] OR "ninety-year"[All Fields] OR ("ninety five"[All Fields] AND "year"[All Fields]) OR "hundred-year"[All Fields] OR "hundred-year"[All Fields] OR "one hundred year"[All Fields] OR "one hundred year"[All Fields]

*Scopus search strategy:*

( ( TITLE-ABS-KEY ( "mobile health application" OR "mobile apps" OR "mobile application" OR "health app" OR "health mobile applications" OR "health care app" OR "health care applications" OR "smartphone app" OR "smartphone applications" OR cellphone* OR “mobile medical apps” OR “mobile medical applications” OR tablet* OR “voice assistant” OR “digital assistant” OR “digital assistants” OR “virtual assistant” OR Alexa OR Siri OR “smart watch” OR “smart watches” OR smartwatch* OR “fitness tracker” OR “fitness trackers” OR “activity tracker” OR “digital health device” OR “wearable devices” OR “digital health technology” OR “digital health technologies” OR “wireless medical device” ) ) AND ( TITLE-ABS-KEY ( "chronic pain" OR "chronic pain management" OR "pain management" OR "pain syndrome" OR "chronic pain syndrome" ) ) ) AND ( ( TITLE-ABS-KEY ( “55 years” OR “55-years” OR “60-years” OR “60 years” OR "65 years" OR "65-years" OR "60-years" OR "70 years" OR "70-years" OR "75 years" OR "75-years" OR "80 years" OR "80-years" OR "85 years" OR "85-years" OR "90 years" OR "90-years" OR "95 years" OR "95-years" OR "100 years" OR "100-years" OR "60 year" OR "65 year" OR "65-year" OR "60-year" OR "70 year" OR "70-year" OR "75 year" OR "75-year" OR "80 year" OR "80-year" OR "85 year" OR "85-year" OR "90 year" OR "90-year" OR "95 year" OR "95-year" OR "100 year" OR "100-year" OR "sixty years" OR "sixty-years" OR "sixty-five years" OR "sixty-five-years" OR "seventy years" OR "seventy-years" OR "seventy-five years" OR "seventy-five-years" OR "eighty years" OR "eighty-years" OR "eighty-five years" OR "eighty-five-years" OR "ninety years" OR "ninety-years" OR "ninety-five years" OR "ninety-five-years" OR "hundred years" OR "hundred-years" OR "hundred years" OR "hundred-years" OR "one-hundred years" OR "one-hundred-years" OR "sixty year" OR "sixty-five year" OR "sixty-five-year" OR "sixty-year" OR "seventy year" OR "seventy-year" OR "seventy-five year" OR "seventy-five-year" OR "eighty year" OR "eighty-year" OR "eighty-five year" OR "eighty-five-year" OR "ninety year" OR "ninety-year" OR "ninety-five year" OR "ninety-five-year" OR "hundred year" OR "hundred-year" OR "one-hundred year" OR "one-hundred-year" ) ) OR ( TITLE-ABS-KEY ( aged OR elder* OR elderly OR "senior citizen" OR senior* OR "oldest old" OR "community dwelling" OR geriatric* OR aging OR retiree* OR pensioner* OR "nursing home" OR "nursing homes" OR "senior center" OR "senior centers" OR "assisted living" OR older OR centenarian* OR octogenarian* OR nonagenarian* OR septuagenarian* OR sexagenarian* OR grandparent* OR grandmother* OR grandfather* OR grandpa* OR grandma* OR gerontol* OR medicare ) ) )

*IEEE search strategy:*

("All Metadata":"mobile health application" OR "All Metadata":"mobile apps" OR "All Metadata":"mobile application" OR "All Metadata":"health app" OR "All Metadata":"health mobile applications" OR "All Metadata":"health care app" OR "All Metadata":“smartphone app") AND ("All Metadata":“chronic pain” OR "All Metadata":“chronic pain management” OR "All Metadata":“pain management” OR "All Metadata":“chronic pain” OR "All Metadata":“pain syndrome OR "All Metadata":“chronic pain syndrome”) AND ("All Metadata":Aged OR "All Metadata":elder* OR "All Metadata":“senior citizen” OR "All Metadata":geriatric* OR "All Metadata":aging OR "All Metadata":older)
