# Supplementary material for: Older Adults’ Pain Outcomes After mHealth Interventions: Scoping Review
Source: JMIR Aging. 2023 May 31;6:e46976. doi: 10.2196/46976 (PMC10267779; doi:10.2196/46976)
Supplement: Multimedia Appendix 2 [file aging_v6i1e46976_app2.docx]

| Author, Location | Study Design and Sample | Device | Intervention Characteristics | Outcomes | Results | Key Conclusions |
| --- | --- | --- | --- | --- | --- | --- |
| Benham et al., 2019  United States | Quasi-experimental pretest-post-test single group study.  N = 12.  Mean age: 70.  Race: 92% white. Diagnoses: Musculoskeletal, neurological pain. | HTC Vive™.  Head-mounted display and 2 hand controllers | Participants selected immersive virtual reality games from an internet based streaming library. Game options: engagement with pets, travel, music, and animals.  Duration: Six weeks. Two sessions/week, 15-45 minutes/session. | Pain intensity: (Numerical Rating Scale) [23]  Depression: (Patient-Reported Outcomes Measurement Information system (PROMIS®) Item Bank v.1.0 – Emotional Distress-Depression [24]  Quality of life: (World Health Organization Quality of Life Scale Brief Version) [25, 26] | Pain intensity: Statistically significant decrease.  Depressive symptoms: decrease was not statistically significant.  Quality of life: no statistically significant changes. | Virtual reality may be feasible for managing or reducing pain in older adults. |
| Dahlberg et al., 2020  Sweden | Longitudinal cohort study.  24-Week Group N = 499. Knee OA: N = 301. Mean age: 64  Hip OA: N = 198.  Mean age: 63.  Race: not specified  0-48 Week  Group  N=138. Knee OA: N = 78. Mean age: 64.  Hip OA: N = 60.  Mean age: 63.  Race: not specified  Diagnoses: OA of knee and hip | Tablet Smartphone  mobile app | Joint Academy® structured and individualized program for people with hip or knee OA.  Included:  Tailored instructions for neuromuscular exercises, with participant ratings.  Text or video information and research on OA  management.  Access to a physiotherapist  Duration: 48-Weeks  Education and quiz: Text or 70 lessons for 48 weeks.  Exercise: Two daily exercises  Physiotherapist chat: continuous. | Pain intensity: (Numerical Rating Scale) [23, 27]  Physical function: (30-second chair stand test) [28] | 24-Weeks  Pain intensity: Decreased monthly by .43 units  Mean knee pain decreased from 5.6 to 3.1.  Mean hip pain decreased from 5.9 to 3.8  Of the participants, 72% percent attained minimal clinically important change in pain, with continuous pain relief in hip and knee.  Physical Function: 30-second chair stand test repetitions increased monthly by .76 repetitions.  Mean increase for participants with knee OA from 10 to 14.3.  Mean increase for participants with hip OA from 10.3 to 14.4  48-Weeks  Pain intensity: Decreased monthly by .39 units.  Mean knee pain decreased from 5.7 to 3.2.  Mean hip pain decreased from 5.8 to 3.8.  Of the participants, 67% attained minimal clinically important change in pain  No statistically significant change in pain reduction between the 24- and 48-week sub-samples.  Physical Function: 30-second chair stand test repetitions increased monthly by .72 repetitions.  Mean repetitions for participants with knee OA increased from 10.3 to 14.4.  Mean repetitions for participants with hip OA increased from 11.1 to 14.9. | Longitudinal use of an app-based OA treatment program was associated with decrease in joint pain, improved physical function.    The app program may be an option for individuals who are unable to participate face-to-face or complement in-person exercise programs to promote long-term adherence. |
| Fanning et al., 2020  United States | Phased Study  (App refinement, then Randomized Control Trial (RCT).  N = 28  Mean age: 70  Race: 82% white  18% black  Diagnosis: Chronic multi-site pain. | Smartphone mobile app | Mobile Intervention to Reduce Pain and improve Health (MORPH)  Including:  Weeks 0-3: in-person weekly group meetings led by a behavioral interventionist and a nutritionist. Instruction on behavior change for diet improvement, movement during pain, pain influencing diet and weight behavior, mind-fulness for diet and weight improvement.  Weeks 4-12:  Group video conferences with instruction on diet change, increasing daily physical activity, pain control, and mindfulness.  Duration: 12-Weeks  Daily activity self-monitoring with a wearable monitor and MORPH app. | Pain intensity (PROMIS 3-item pain intensity scale) [29]  Pain interference (PROMIS 8-item pain interference scale) [29]  Physical function (Short Physical Performance Battery)  Weight  Physical activity:  Daily steps, minutes of sedentary time, postural shifts (ActivPAL^TM^ device worn on the thigh, Fitbit Alta device worn on wrist) | Pain intensity: moderate-to-large effect, trending the intervention.  Pain interference:  No clinically meaningful difference, small effect, trending the intervention.  Physical function: clinically meaningful difference, trending the intervention.  Weight  Large effect in the intervention group  Physical activity:  Fitbit  Moderate-to-large effect on average daily steps trending the intervention group (757 more steps).  Moderate effect on breaks, in the control group (5 more breaks).  ActivPAL  Small effect on -recorded daily steps, trending the intervention group (298 more daily steps).  Moderate effect on sedentary time, trending the intervention group (65 minutes less) | A multi-component intervention mostly home delivered that included app monitoring may be feasible for older adults, with positive effects on pain intensity, clinically meaningful effects on physical function, weight loss and physical activity. |
| Gohir et al., 2021  United Kingdom | RCT  N = 105  Mean age:  65 intervention and 68 usual care self-management  Race: not specified  Diagnoses: OA of knee | Smartphone mobile app | iBEATOA program  App delivered structured/tailored exercise and OA information.  OA educational sessions with a quiz  Adherence encouraged by daily emails, smartphone notifications, or by the physiotherapist.  Duration: Six Weeks Daily | NRS: Pain intensity  OA-related pain, stiffness, and physical function (Western Ontario and McMaster Universities Osteoarthritis Index (WOMAC)) [30]  Physical functioning (-second sit-to-stand test, [31] Timed Up-and-Go test [32])  Symptoms and quality of life (Arthritis Research UK Musculo-skeletal Health Questionnaire) [33]  Maximum voluntary contraction of quadriceps and hamstring muscles (isokinetic peak torque) [34, 35]  Quantitative sensory testing: pressure pain threshold [36]; temporal summation [37]; conditional pain modulation [38] | Pain: Significant improvement in pain scores in the intervention group between baseline and 6 weeks but, not in the usual care group.  OA-related pain, stiffness, and physical function (WOMAC):  Intervention group improved more than the control group.  Physical function:  Intervention group improved in the 30-second sit-to-stand test and Timed Up-and Go test compared with the control group.  No statistically significant between-group differences in other measures of the Arthritis UK Musculoskeletal Health Questionnaire scores.  Hamstring isokinetic strength at 60°/s Intervention group increased more than in the control group  No statistically significant between group differences in quantitative sensory testing. | App delivered education on OA may improve pain and pain-related outcomes in patients when compared to usual care. |
| Kaul et al., 2022  United States | Pilot RCT  N = 31.  Mean age: 67  Race: 45% white, 42% black, 13% other  Diagnosis: Chronic non-cancer pain | Tablet Smartphone  mobile app | Mymee app  combines self-monitoring, tracking and tailored coaching.  Participants tracked pain symptom, dietary intake, exercise, sleep, and behaviors that triggered or reduced pain. Structured/tailored telephone coaching sessions for pain management. Coaching based on social cognitive theory and motivational interviewing.  Duration: 12-Weeks  Daily tracking  Weekly coaching up to 30 minutes for up to 12 sessions maximum. | Pain intensity (NRS)  Pain-related disability (Roland-Morris Disability Questionnaire) [39]  Pain self-efficacy (Pain Self-Efficacy Questionnaire) [40]  Anxiety (General Anxiety Disorder scale) [41]  Positive and negative emotions (Positive and Negative Affect Scale) [42]  Quality of life (Quality-of-Life Enjoyment and Satisfaction Questionnaire) [43] | Pain intensity: Decrease in the intervention group compared with control.  Pain-related disability: Decrease in the intervention group compared with control.  Pain self-efficacy: Increase in the intervention group compared with control.  Anxiety: Decreased, in the intervention group compared with control.  Positive and negative emotions: non-significant difference between the groups.  Quality of life: non-significant difference between the groups. | Multicomponent intervention with the Mymee app and coaching may have preliminary efficacy on pain symptoms.  The sample small to determine differences between the study groups and usability of the app may have impacted the information available for coaching. |
| Piette et al., 2022  United States | Randomized non-inferiority comparative effectiveness trial  N = 278  Mean age = 64 Race: 80.1% white; 16% black intervention, and 84.4% white; 9% black control  Diagnosis: Chronic back pain. | Artificial Intelligence (AI)  Personalized suggestions based on a matrix of patient characteristics and experiences. | AI Cognitive behavioral therapy for chronic pain, (AI-CBT-CP)  Intervention: weekly patient feedback via daily interactive voice response calls generated by an AI engine.  Duration: 10-Weeks  CBT 15- or 45-minute live therapist session or an asynchronous individualized interactive voice response therapist message.  Control group: 10 therapist-delivered telephone CBT-chronic pain sessions (45 minutes/session). | Pain-related disability (Roland-Morris Disability Questionnaire) [39]  Pain intensity (Numeric Rating Scale)  Pain-related interference (Brief Pain Inventory) [44,45]  Depressive symptoms (Patient Health Questionnaire) [46]  Health-related quality of life (Veterans Short Form-12) [47]  Patients’ overall impression of change since starting treatment (Patient Global Impression of Change) [44] | Pain-related disability, at 3 months mean difference between the intervention and control at three months was significant: -.72 points. At 6 months: there was non-inferiority and marginal superiority of AI-CBT-CP to live telephone CBT-CP.  More intervention participants had clinically meaningful improvements at 6 months in pain-related disability compared to the standard CBT-CP (37% vs. 19%, *p* = .01)  Intervention participants improved in pain intensity at six months compared to the standard CBT-CP.  No statistically significant difference between all other outcomes at three and six months, demonstrating non-inferiority of AI-CBT-CP to live telephone CBT-CP | AI-CBT was non-inferior to telephone CBT-CP and required less therapist time. |

*Note.* CI – confidence interval. AI – artificial intelligence. CBT – cognitive behavioral therapy. CP – chronic pain. OA – osteoarthritis.
